# Supplementary material for: Iodine Biofortification of Dandelion Plants (Taraxacum officinale F.H. Wiggers Coll.) with the Use of Inorganic and Organic Iodine Compounds
Source: Molecules. 2023 Jul 25;28(15):5638. doi: 10.3390/molecules28155638 (PMC10419995; doi:10.3390/molecules28155638)

Supporting information file

Iodine biofortification of dandelion plants (*Taraxacum officinale* F.H. Wiggers coll.) with the use of inorganic and organic iodine compounds

Iwona Ledwożyw-Smoleń[1*], Joanna Pitala[2], Sylwester Smoleń[1,2], Marta Liszka-Skoczylas[3], Peter Kováčik[4]

1 Faculty of Biotechnology and Horticulture, University of Agriculture in Kraków, al. Mickiewicza 21, 31-120 Kraków; sylwester.smolen@urk.edu.pl;

2 Laboratory of Mass Spectrometry, University of Agriculture in Kraków, al. Mickiewicza 21, 31-120 Kraków; joanna.pitala@urk.edu.pl

3 Department of Engineering and Machinery for Food Industry, Faculty of Food Technology, University of Agriculture in Krakow, al. Mickiewicza 21, 31-120 Krakow, Poland; marta.liszka-skoczylas@urk.edu.pl

4 Department of Agrochemistry and Plant Nutrition, Institute of Agronomic Sciences, Faculty of Agrobiology and Food Resources, Slovak University of Agriculture in Nitra, Tr. A. Hlinku 2, 949 01 Nitra, Slovakia; peter.kovacik@uniag.sk

* Correspondence: iwona.ledwozyw-smolen@urk.edu.pl

Table S1 Precursor/product transitions of analytes at LC-MS/MS technique: salicylic acid (SA); 5-iodosalicylic acid (5-ISA); 3,5-diiodosalicylic acid (3,5-diISA) and jasmonic acid (JA)

Analyte	Precursor ion (m/z)	Product ion (m/z)	transition
Salicylic acid (SA)	136.8	93.1	quantifying
Salicylic acid (SA)	136.8	64.9	qualifying
5-iodosalicylic acid (5-ISA)	262.9	126.7	quantifying
5-iodosalicylic acid (5-ISA)	262.9	218.8	qualifying
3,5-diiodosalicylic acid (3,5-diISA)	388.8	126.7	quantifying
3,5-diiodosalicylic acid (3,5-diISA)	388.8	344.6	qualifying
Jasmonic acid (JA)	209.15	58.77	quantifying
Jasmonic acid (JA)	209.15	165.0	qualifying

Figure S1. The 5-ISA calibration curve and 5-ISA standard peaks at 1, 5, 10, 50 and 100 ng 5-ISA·ml^{-1}.

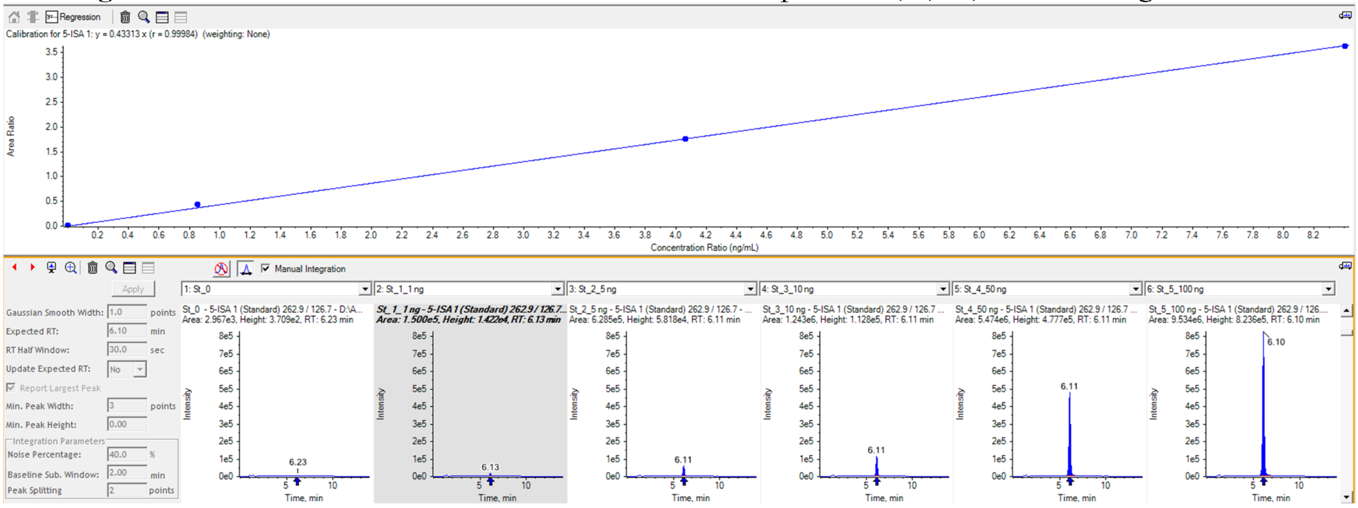

Equal Y-axis scale fitted to the highest concentration standard of 5-ISA

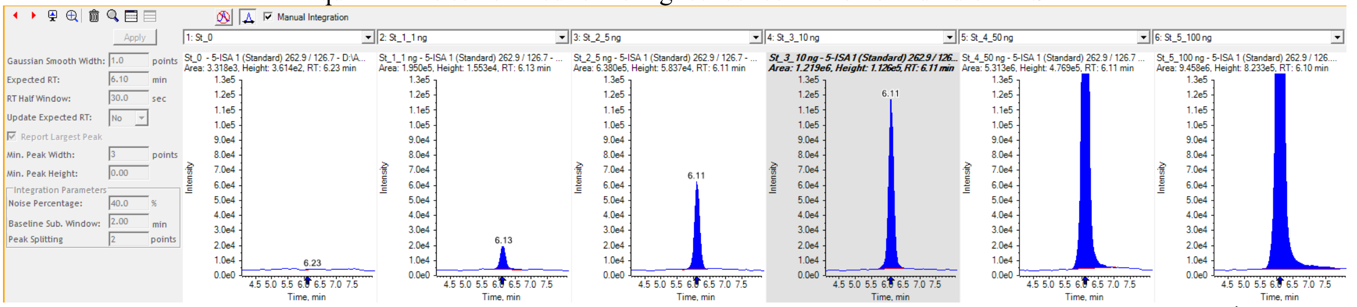

Zooming - the same scale of the Y axis adjusted to the standard with a concentration of 10 ng 5-ISA·ml^{-1}.

Figure S2. The 3,5-diISA calibration curve and 3,5-diISA standard peaks at 1, 5, 10, 50 and 100 ng 3,5-diISA·ml^{-1}.

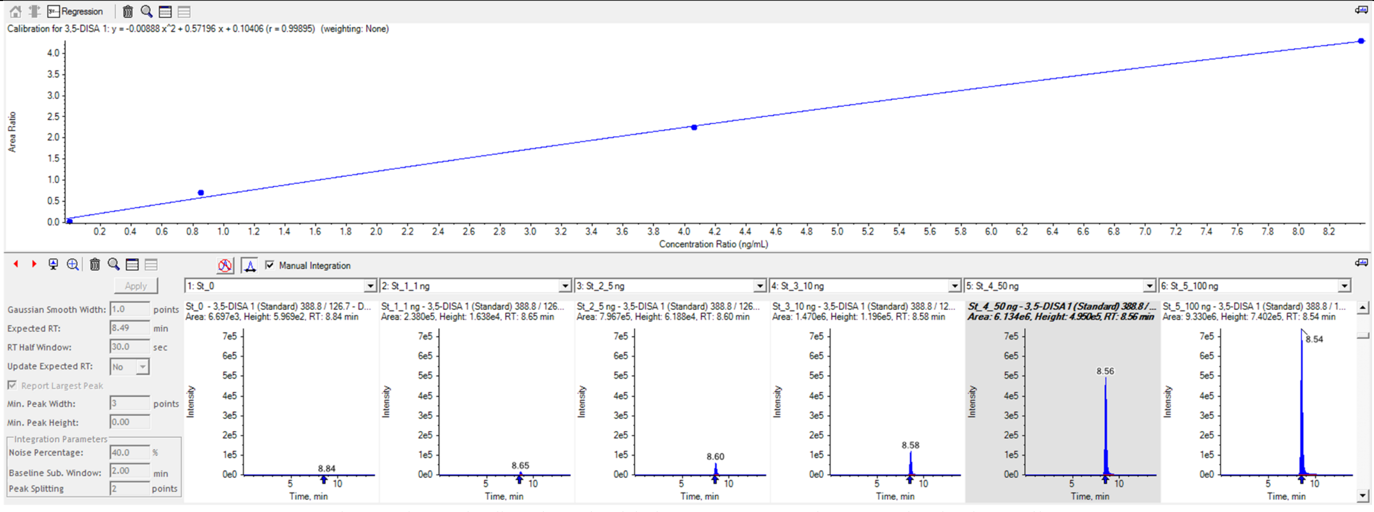

Equal Y-axis scale fitted to the highest concentration standard of 3,5-diISA

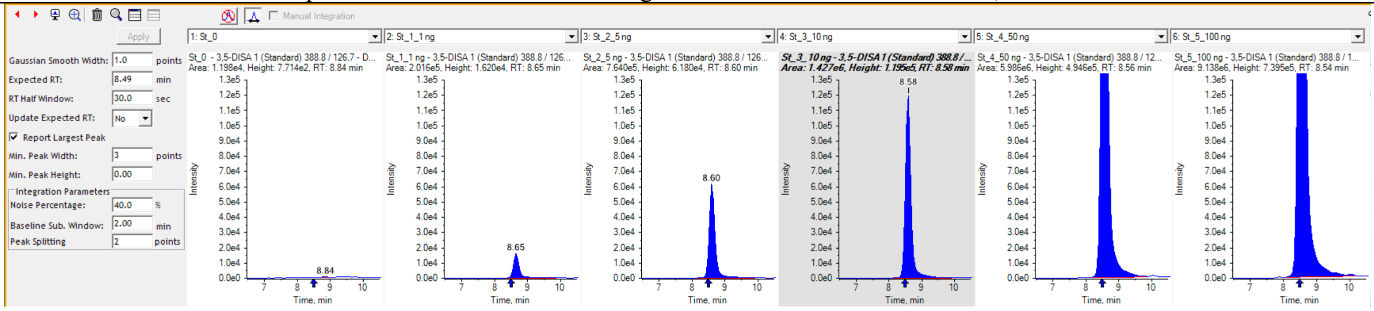

Zooming - the same scale of the Y axis adjusted to the standard with a concentration of 10 ng 3,5-diISA·ml^{-1}.

Figure S3. Identification example by pairs of MRM and retention time:

for 5-ISA	for 3,5-diISA
Below:<br>first row - 1 pair of quantitative MRM (262.9/126.7);<br>second row - 2 MRM pair - qualitative (262.9/218.8)<br>for leaves (left) and roots samples (right)	Below:<br>first row - 1 pair of quantitative MRM (388.8/126.7);<br>second row - 2 MRM pair - qualitative (388.8/344.6)<br>for leaves (left) and roots samples (right)

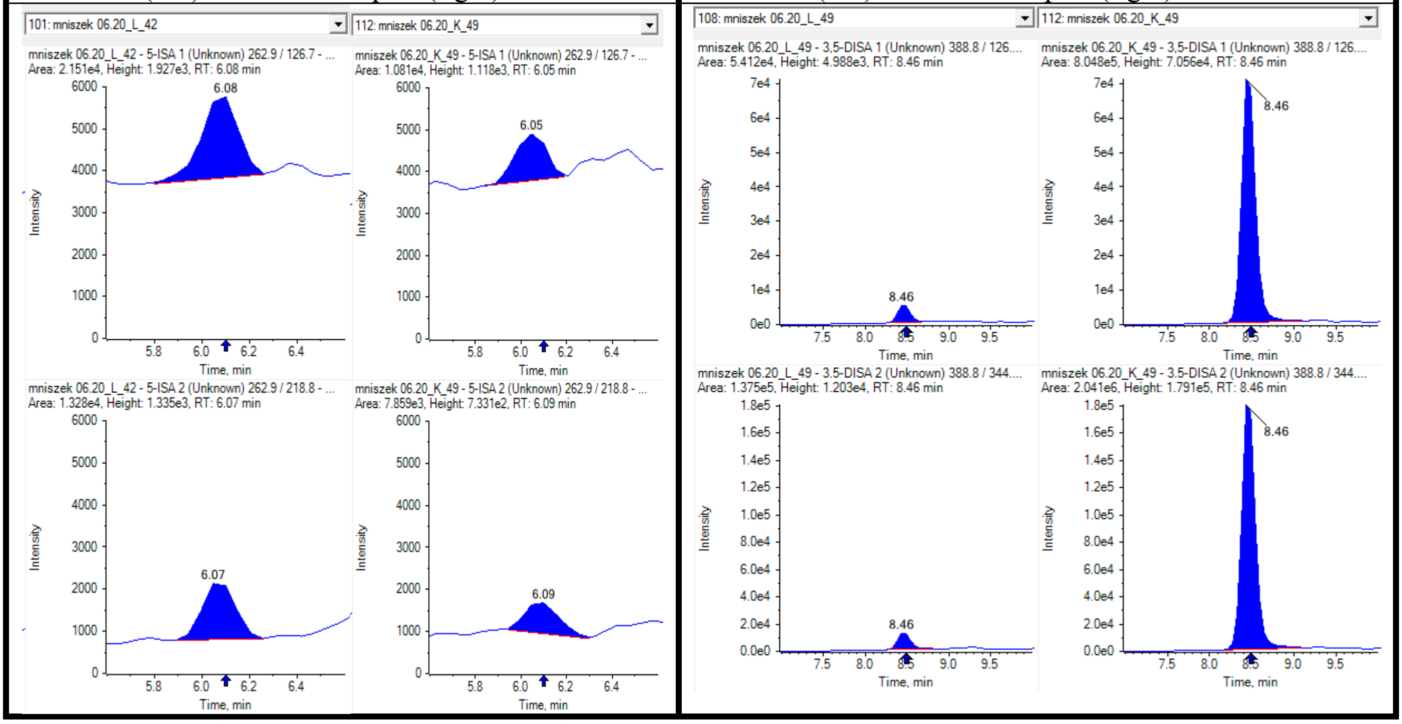

Supplement: Supplementary file 1 [file molecules-28-05638-s001.zip › molecules-2502739-supplementary.pdf]
